# Progress of Molecular Targeted Therapies for Advanced Renal Cell Carcinoma

**DOI:** 10.1155/2013/419176

**Published:** 2013-09-04

**Authors:** Alessandro Conti, Matteo Santoni, Consuelo Amantini, Luciano Burattini, Rossana Berardi, Giorgio Santoni, Stefano Cascinu, Giovanni Muzzonigro

**Affiliations:** ^1^Department of Clinical and Specialist Sciences, Urology, Polytechnic University of the Marche Region, AOU Ospedali Riuniti Umberto I-GM Lancisi and G Salesi, Ancona, Italy; ^2^Medical Oncology, Polytechnic University of the Marche Region, AOU Ospedali Riuniti Umberto I-GM Lancisi and G Salesi, Ancona, Italy; ^3^School of Pharmacy, Section of Experimental Medicine, University of Camerino, Italy

## Abstract

Vascular endothelial growth factor (VEGF) plays a crucial role in tumor angiogenesis. VEGF expression in metastatic renal cell carcinoma (mRCC) is mostly regulated by hypoxia, predominantly via the hypoxia-induced factor (HIF)/Von Hippel-Lindau (VHL) pathway. Advances in our knowledge of VEGF role in tumor angiogenesis, growth, and progression have permitted development of new approaches for the treatment of mRCC, including several agents targeting VEGF and VEGF receptors: tyrosine kinase pathway, serine/threonine kinases, **α**5**β**1-integrin, deacetylase, CD70, mammalian target of rapamycin (mTOR), AKT, and phosphatidylinositol 3′-kinase (PI3K). Starting from sorafenib and sunitinib, several targeted therapies have been approved for mRCC treatment, with a long list of agents in course of evaluation, such as tivozanib, cediranib, and VEGF-Trap. Here we illustrate the main steps of tumor angiogenesis process, defining the pertinent therapeutic targets and the efficacy and toxicity profiles of these new promising agents.

## 1. Introduction

The development of targeted agents has completely changed the therapeutic landscape of metastatic renal cell carcinoma (mRCC). Starting from sorafenib in 2005, several agents have been sequentially approved by the US Food and Drug Administration (FDA), such as sunitinib, bevacizumab plus interferon, everolimus, temsirolimus, pazopanib, and at last axitinib. Nevertheless, despite recent success, complete responses to antiangiogenic therapies are rare, and patients do progress in response to antivascular endothelial growth factor (VEGF) targeted therapies. The increasing awareness of the molecular mechanisms underlying tumor angiogenesis in RCC parallels the development of novel antiangiogenic agents, mainly directed against VEGF-A, platelet-derived growth factor (PDGF) receptors (PDGFRs), and VEGF receptor-2 (VEGFR-2), with a list of novel targets currently in course of evaluation.

In RCC, the loss of 3p induces a high incidence of Von Hippel-Lindau (VHL) tumor suppressor gene inactivation, which promotes hypoxia-inducible factor-1 (HIF-1) dysregulation [1], and leads to increased expression of VEGF and other proangiogenic factors [2]. HIF-1 activity is essential for genetic stability, proliferation, migration, and survival, contributing also to tumor cell metabolism and drug and radiation resistance [3, 4]. 

**Figure 1:**
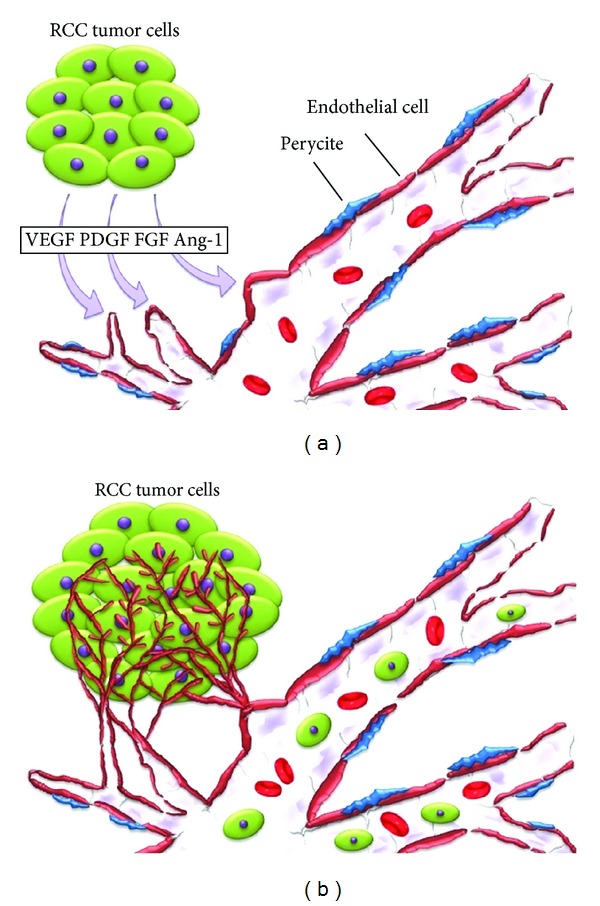
RCC tumor angiogenesis. (a) Factors influencing promotion of tumor angiogenesis. (b) Tumor vessel sprouting and metastasis. Ang-1; angiopoietin-1; FGF; fibroblast growth factor; PDGF; platelet derived growth factor; RCC; renal cell carcinoma; VEGF; vascular endothelial growth factor.

Tumor angiogenesis is the result of the interaction among several components of tumor microenvironment. This multistep process involves the migration and proliferation of endothelial cells (ECs), the recruitment of hematopoietic progenitor cells (HPCs), highly proliferative circulating endothelial cells (CECs), and endothelial progenitor cells (EPCs), the detachment and migration of pericytes, and the cooption of neighbouring preexisting vessels [5]. Several signaling pathways are involved in tumor angiogenesis, such as VEGF, which plays a crucial role in RCC carcinogenesis [6, 7], PDGF, the mammalian target of rapamycin (mTOR), VHL, a tumor suppressor gene, and HIF (Figure 1). The transit from an anti- to a proangiogenic phase, also called angiogenic switch, leads to the rapid formation of blood vessels [8]. The insufficient vascularisation accompanying rapid tumor growth leads to hypoxia [9, 10].

VEGF promotes EC migration and proliferation, thus stimulating extracellular matrix (ECM) and EC basement membrane digestion by plasminogen activator (PA) and metalloproteinases (MMPs). Tumor vessel sprouting requires, at first, the removal of pericytes from preexisting blood vessels. As a result of this process, ECs begin to proliferate and migrate to form new vessels, subsequently surrounded by mesenchymal cells differentiated into pericytes. Tumor cell-derived VEGF induces also the recruitment of CECs and EPCs. CECs are generally accepted as cells expressing the von Willebrand factor (vWf), CD146, CD34, and VE-cadherin. Their clinical significance is still under investigation. Recent studies have observed increased levels of CECs in RCC patients treated with sunitinib, likely caused by the drug targeting of immature tumor vessels [11]. EPCs express CD133, CD34, and VEGFR-2 [12]. Circulating EPCs begin to differentiate by loosing the CD133 marker and gaining EC-specific markers such as vWf, CD31, and VE-cadherin [13], thus enhancing tumor development and growth [14]. In 2003, Rafii et al. observed that MMP-9 was capable of mobilizing EPCs [15]. Their migration is mediated by the chemokine stromal-cell-derived factor-1(SDF-1), which is upregulated during hypoxia [16].

A related circulating cell population is the HPC, expressing VEGFR1. Along with EPCs expressing VGFR2, they colonize the sprouting vessel connective tissue and also provide growth factors and cytokines to create a microenvironment favouring the start of metastatic process, in which tumor cells can localize and proliferate [17]. Recently, Powles et al. have demonstrated the association between increased numbers of circulating HPCs and poor outcome in patients with mRCC treated with sunitinib [18].

The complete formation of a lumen in novel sprouting vessels needs the contribution of stalk cells, stimulated by VEGF, ECM, and EC-derived EGF-like domain 7 (Egfl7), which promotes the separation and proper spatial arrangement of the angioblasts and allows subsequent assembly of vascular tubes [19].

The detachment of pericytes during tumor angiogenesis is mediated by VEGF and angiopoietin-2 (Ang-2)/type I tyrosine kinase receptor 2 (TIE 2). The moved pericytes are recruited by PDGF expressed in ECs, surrounding and stabilizing vessel sprouts [20]. 

The expression of angiogenic factors in RCC is also regulated by VHL-independent signaling pathways, such as the oncoprotein homologue of the mouse double minute 2 (HDM2) that leads to the constitutive expression of HIF**α**. HDM2 also regulates the protein levels of HIF angiogenic targets, such as VEGF, PA inhibitor-1 (PAI-1), and endothelin-1 (ET-1) [21].

Therefore, in this review we illustrate the progress of molecular targeted therapy, describing the emerging agents in course of evaluation and the future directions in the treatment of RCC patients. 

## 2. Tyrosine Kinase Inhibitors “Enlarged” Family

In the last two years, the list of novel anti-angiogenetic agents under evaluation has progressively enlarged, including now axitinib, tivozanib, dovitinib, cediranib, linifanib, volociximab, regorafenib, and other new emerging molecules [22]. In January 2012, axitinib was approved in the second line treatment of mRCC patients. Axitinib is an oral inhibitor of VEGFR, PDGFR, and c-kit, and it may afford a relevant contribution for the future management of RCC patients. In a phase II trial enrolling 62 treatment-refractory RCC patients progressed on sorafenib and treated with oral 5 mg axitinib twice daily, the median progression-free survival (PFS) was 7.4 months. Grades 3 to 4 adverse events included hand-foot syndrome (16.1%), fatigue (16.1%), hypertension (16.1%), dyspnea (14.5%), diarrhea (14.5%), dehydration (8.1%), and hypotension (6.5%) [23]. In addition, another phase II trial with axitinib, enrolling cytokine-refractory nephrectomised patients, showed response rate (RR) of 44.2% and a median time to progression (TTP) of 15.7 months [24]. The AXIS phase III trial, comparing the effectiveness of axitinib and sorafenib in pretreated advanced RCC patients, has shown a significant median PFS (6.7 versus 4.7 months), with a more pronounced benefit for patients after immunotherapy (12.1 versus 6.5 months) [25]. With regard to the front-line use of axitinib, the preliminary results shown at the 2012 annual meeting of the American Society of Clinical Oncology (ASCO) from the randomized phase II study AGILE 1046 demonstrated a median PFS of more than 1 year and an overall response rate of 40.2% for axitinib [26]. Furthermore, several trials are presently ongoing to evaluate the role of axitinib as neoadjuvant therapy (NCT01263769), in combination with everolimus (NCT01334073), or coadministered with PF-04856884, a selective angiopoietin (Ang)-2 inhibitor (NCT01441414). 

Among the emerging agents, tivozanib (AV-951) has been demonstrated to be effective in the first line setting of mRCC patients with prior nephrectomy [27]. Tivozanib is a potent inhibitor of VEGFR-1, 2, and 3, cKit, and PDGFR. Hypertension (50.0%) and dysphonia (21.7%) were the most commonly reported treatment-related adverse events of any grade. There was a low incidence of diarrhea (12.1%), fatigue (8.1%), stomatitis (4.4%), and hand-foot syndrome (3.7%). In the phase III “TIVO-1” study [28], a total of 517 patients were randomized to tivozanib (*N* = 260) or sorafenib (*N* = 257) in first line advanced RCC patients. Tivozanib showed a statistically significant improvement in median PFS compared to sorafenib (11.9 versus 9.1 months) in the subpopulation of patients who were pretreated with systemic therapy including cytokines. In patients who were treatment naïve (70% of total study population), tivozanib showed a statistically significant improvement in PFS, with a median PFS of 12.7 months compared with 9.1 months for sorafenib (HR 0.756, 95% CI 0.580–0.985; *P* = 0.037). Tivozanib demonstrated favorable tolerability, with a lower rate of dose interruptions (18% versus 35%, *P* < 0.001) and reductions (14% versus 44%, *P* < 0.001). The most common ≥grade 3 adverse events (AEs) due to tivozanib compared to sorafenib were hypertension (25% versus 17%), hand-foot syndrome (2% versus 17%), diarrhea (2% versus 6%), fatigue (5% versus 4%), and neutropenia (2% versus 2%). While the progression-free survival was improved, the overall survival (OS) showed a trend toward a detrimental effect with the tivozanib arm with a median OS of 28.8 months versus 29.3 months in the sorafenib arm based on the pre-new drug application (NDA) meeting with the US Food and Drug Administration (FDA) [29] which later led to the FDA ODAC meeting to disapprove tivozanib as an indication for RCC.

A phase I study has been completed to evaluate the safety of tivozanib in combination with temsirolimus in subjects with mRCC (NCT00563147). 

With regard to the third line treatment of mRCC patients, dovitinib seems to represent a valid option. It is a fibroblast growth factor receptor (FGFR) and VEGFR inhibitor, presently in course of evaluation in a phase III trial (NCT01223027). The most common adverse events shown in the phase I/II study were nausea (80%; G3:5%), diarrhea (70%), vomiting (65%), asthenia (50%; G3:15%), anorexia (45%; G3:5%), headache (30%; G3:5%), hypertension (25%; G4:5%), and rash (23%; G3:5%). In a phase II trial enrolling 59 previously treated patients, dovitinib was administered with a dose schedule of 500 mg/day 5 days on/2 days off. In this study, PFS and OS were 6.1 and 16 months, respectively [30]. Results are awaited from a phase III trial (NCT01223027) enrolling 550 patients who must have received one VEGF-targeted therapy and one prior mTOR inhibitor therapy to evaluate dovitinib versus sorafenib in the third line setting of mRCC treatment.

Recent advances in understanding the role of fibroblast growth factor 2 (FGF2) and FGF receptor (FGFR) in modulating resistance to sunitinib [31] led to the development of PD173074, a reversible FGFR and VEGFR inhibitor. Thus, FGF2 supports endothelial proliferation and de novo tubule formation in the presence of sunitinib, suppressing sunitinib-induced retraction of tubules. Currently, several studies are analyzing the efficacy and safety of PD173074 in small cell lung cancer and RCC. 

At this time, the list of emerging TKIs under study in phase II trials includes cediranib, linifanib, regorafenib, brivanib, vandetanib, lenvatinib, and several other agents. Cediranib (AZD2171) is an oral inhibitor of VEGFR1-3, PDGFR*β*, and c-kit. In 2012, Mulders et al. have published the results from a phase II trial (NCT00423332) in 71 previously untreated mRCC patients randomized to receive cediranib (*n* = 53) or placebo (*n* = 18). They revealed 34% PR and 47% stable disease (SD), and cediranib was generally well tolerated [32]. Furthermore, another phase II trial (COSAK) is ongoing to assess the efficacy of cediranib 30 mg versus cediranib 30 mg plus 175 mg saracatinib (AZD0530), an Src Family oral inhibitor, in patients with relapsed metastatic clear cell RCC (ccRCC). 

Linifanib (ABT-869) is a potent inhibitor of VEGFR, PDGFR, fms-like tyrosine kinase 3 (FLT3), c-kit, and colony stimulating factor-1 receptor (CSF1R). In 2012, Tannir et al. have published their results [33] from an open-label multicenter trial (NCT00486538) in 53 patients previously treated with sunitinib, receiving oral linifanib 0.25 mg/kg (12.5–25.0 mg) daily. They showed 13.2% overall RR, with a median PFS and OS of 5.4 and 14.5 months, respectively.

Regorafenib (BAY 73-4506) is an orally multikinase inhibitor targeting VEGFR, c-kit, RET, FGFR, PDGFR, and serine/threonine kinases (RAF and p38MAPK). A phase II trial (NCT00664326) on 33 patients treated with BAY 73-4506 160 mg once daily on a 3-week on/1-week off schedule showed 27% PR and a 42% SD [34].

Brivanib and vandetanib represent two more members of the VEGF-related antiangiogenic family. Brivanib is an oral, dual VEGFR-2 and FGFR-1 tyrosine kinases inhibitor. A phase II, open-label investigation conducted to assess is activity in mRCC patients has been opened in November 2010 (NCT01253668). On the other hand, vandetanib, also known as ZD6474, is an antagonist of VEGFR and EGFR. A phase II trial (NCT01372813) has been terminated for insufficient accrual. 

In 2006, Jermann et al. [35] published the results of a phase II trial of gefitinib, a low-molecular-weight epidermal growth factor receptor (EGFR) TKI, in patients with locally advanced, metastatic, or relapsed RCC. They did not observe objective responses (OR), although 14 patients (53.8%) had SD. Treatment was generally well tolerated. Furthermore, gefitinib was also evaluated in combination with pegylated IFN*α*, without obtaining significant results [36].

The development of XL999 and tandutinib was stopped due to their toxicity profiles. In 2006, a phase II trial (NCT00277316) was opened to investigate the activity of XL999, a multiple inhibitor of VEGFR, PDGFR, FGFR, FLT-3, and Src. The employment of this molecule was stopped due to the cardiac AEs revealed in the participants of this study. In the same year, Shepard et al. [37] evaluated the efficacy and tolerability of tandutinib, an oral inhibitor of type III tyrosine kinase receptor kinases, observing absence of clinical activity and excessive toxicity in these patients. 

Finally, the list of novel TKI agents presently under study also includes crizotinib, an anaplastic lymphoma kinase (ALK), and c-Met TKI (CREATE, NCT01524926) and BIBF 120, which is under evaluation versus sunitinib in untreated mRCC patients (NCT01024920).

## 3. Anti-VEGFR Dual Strategy: mAb and Derivatives

Since the approval of bevacizumab in 2009 for the treatment of RCC patients, several VEGF/VEGFR blocking agents have been developed, such as VEGF-Trap and ramucirumab. VEGF-Trap (AVE0005, ziv-aflibercept) is a VEGF-blocking agent with both higher affinity and activity than bevacizumab [38]. It is a derivative of VEGFR1 and VEGFR2 [38], with minimal interactions with extracellular matrix, and this property may be the key to its satisfying pharmacokinetic profile. At present, a phase II trial (NCT00357760) is in course in patients who must have received at least one prior treatment with a TKI for metastatic ccRCC. VEGF-Trap (ziv-aflibercept). The main side effects revealed in the phase I trial were proteinuria (37%), fatigue (32%), injection site reactions (18%), nausea (17%), myalgia and anorexia (16% each), hypertension (13%), and voice hoarseness (11%), with 7% of grades 3-4 events [39]. 

Ramucirumab (IMC-112-1B) is a fully human IgG1 mAb targeting VEGFR-2, used in a phase II trial (NCT00515697). The results obtained, however, (49% SD lasting over 5 months, 6 months PFS) must be confirmed in randomized trials on TKI-refractory mRCC patients.

## 4. Targeting mTor Pathway at Several Levels: mTor, AKT, and PI3K Inhibitors

Recent strategies against mTor pathway include the use of selective inhibitors of mTOR, AKT, or PI3K and dual PI3K/mTOR inhibitors. Between the emerging mTOR inhibitors, ridaforolimus (AP23573) [40] and AZD8055 [40] have been demonstrated to be active in RCC patients. Ridaforolimus is an mTOR complex 1 (MTORC1) selective inhibitor that has been studied in phase I trial [39] administered as a 30-minute intravenous infusion once daily for 5 consecutive days every 2 weeks (QDx5) in a 28-day cycle. On the basis of this study, a dose of 12.5 mg/d is being evaluated in phase II trials. Ridaforolimus is currently under evaluation even in combination with vorinostat, an HDAC inhibitor (NCT01169532), and with AKT inhibitor MK2206 or *γ*-secretase inhibitor MK-0752 in patients with advanced solid tumors (NCT01295632).

On the other hand, AZD8055, a dual mTORC1 and mTORC2 inhibitor, has been tested in a phase I clinical trial at different doses (NCT00731263), showing a maximum tolerated dose (MTD) of 90 mg BID [41].

Moreover, perifosine, an AKT and MAP kinase inhibitor, has been evaluated in a phase II trial (NCT00448721) in 24 patients who had experienced disease progression after receiving either sorafenib or sunitinib. This study revealed 8% PR, 42% SD at 12 weeks, and a median PFS pf at 19 weeks. The most common grades 3 and 4 adverse events were dyspnea (8%), hyponatremia (8%), pulmonary embolism (4%), and arthralgia (4%) [42]. Two separate phase I trials have investigated its use in combination with sunitinib (NCT00399152) or sorafenib (NCT00398814), observing in both cases a good tolerability and promising results in stabilizing kidney cancer. The efficacy of MK2206, another AKT inhibitor, is presently under evaluation in a phase II trial (NCT01239342) versus everolimus in refractory RCC patients. 

Concerning PI3K inhibitors, BKM-120 is presently under evaluation in several clinical trials, administered alone or in combination with bevacizumab in patients who have failed at last one prior anti-VEGF therapy (NCT01283048).

Finally, this family also includes dual PI3K/mTOR inhibitors, such as NVP-BEZ235 and GDC-0980. NVP-BEZ235, a dual PI3K/mTOR inhibitor, has been demonstrated to reduce in vitro cell proliferation by inducing nuclear translocation of p27 and to downregulate Akt, Mnk-1, eIF4E, and 4EBP-1 phosphorylation and cyclin D1 and HIF2*α* compared to rapamycin [43]. In July 2011, Roulin et al. evaluated in vitro the combination between NVP-BEZ235 and sorafenib, resulting in reduced tumour cell proliferation and increased tumour cell apoptosis in vitro [44]. Moreover, NVP-BEZ235 is under study with everolimus in a phase I trial (NCT01482156). With regard to GDC-0980, it is under evaluation in comparison with everolimus in mRCC patients progressed on VEGF-targeted therapy (NCT01442090).

## 5. Novel Emerging Targets and Future Directions

The role of angiopoietin/Tie2 pathway in tumor angiogenesis has led to the development of several inhibiting agents, such as AMG 386 and PF-4856884. Their action alone or in combination with TKIs is subject of ongoing clinical trials. The efficacy and tolerability of trebananib (AMG 386) plus sorafenib have been tested in 152 ccRCC patients receiving sorafenib 400 mg orally twice daily plus intravenous AMG 386 at 10 mg/kg (arm A) or 3 mg/kg (arm B) or placebo (arm C) once weekly. PFS was not significantly different in three groups, which all revealed high frequency of AEs, such as diarrhea (70% in patients receiving 10 mg/kg AMG 386 and 67% in those receiving 3 mg/kg), hand-foot syndrome (52 and 47%), alopecia (50 and 45%), and hypertension (42 ad 49%) [45]. In addition, AMG 386 has been evaluated in combination with sunitinib in 85 mRCC patients naïve to angiogenesis inhibitors, treated with sunitinib 50 mg PO QD (4 weeks on, 2 weeks off) plus AMG 386 at 10 mg/kg (A) or 15 mg/kg (B). Median OR rate (ORR) was 58% in group A, including 1 CR, and 59% in group B. This combination appeared to be tolerable, with 16% (A) and 29% (B) AMG 386 discontinuations due to AEs [46]. At present, AMG-386 is under studying also in combination with temsirolimus (NCT01548482). 

Recent advances concerning the role of integrin receptors in tumor angiogenesis have led to the development of volociximab. It is a high-affinity anti-*α*5*β*1 integrin mAb, able to avoid *α*5*β*1 integrin binding to fibronectin in the ECM, thus inducing apoptosis of ECs [47]. The results coming from a phase II study (NCT00100685) in 40 patients with clear cell mRCC showed a stabilizing activity of volociximab, with only one PR and 32 SD observed. Most frequent side effects were fatigue (67.5%), nausea (35%), dyspnea (20%), and arthralgia (17.5%), with no serious side effects observed [48]. 

The use of antibody-drug conjugate (ADC) has opened novel landscapes for the treatment of RCC patients. ADC structure is composed a mAb, able to recognize and bind specific antigens on RCC cell surfaces, and a pro-drug activated by mAb-antigen interaction. Lymphocyte activation antigen CD70 that is expressed on RCC cells is one of the main targets of this new strategy. The therapeutic role of CD70 depends on the possibility of conjugating pro-drugs with the anti-CD70 mAb. The interaction between anti-CD70 mAb and CD70 on tumor cell surfaces led to prodrug release and activation. MDX-1203 is an mAb directed against the human CD70 molecule conjugated with an analogue of CC-1065 (rachelmycin) as prodrug. Its antitumor activity is due to the alkylating action of CC-1065 on adenine, leading to a reduced proliferation of CD70+ tumor cells [49]. MDX-1203 is actually in course of evaluation in a phase I study (NCT00944905) in patients with advanced/recurrent clear cell RCC. Moreover, MDX-1411 and SGN-75, two anti-CD70 Ab-drug conjugates, are in course of evaluation in pretreated ccRCC patients (NCT00656734 and NCT01015911, resp.).

A different approach consists in the use of radio-labeled mAb against antigens expressed on RCC cells surface. The G250 antigen has been identified as carbonic anhydrase isoenzyme 9 (MN/CA IX). It is expressed in over 95% of RCC cells and it is absent in normal kidney [50]. The radio-labeled mAb targeting G250 is currently employed in several clinical trials. Recently, the chimeric mAb cG250 has been demonstrated in in vitro studies to induce antibody-dependent cellular cytotoxicity (ADCC) against G250^+^ RCC cells [51]. The employment of G250 in the diagnosis and treatment of RCC is under evaluation. 

Histone deacetylase (HDAC) determines the acetylation status of histones, thus regulating gene expression. In 2004, Shao et al. reported that aberrant HDAC activity plays a relevant role in carcinogenesis [52]. HDAC catalyses the removal of acetyl groups from N-acetyl lysine on a histone. The effects of HDAC inhibitors include the activation of cyclin-dependent kinase inhibitor p21WAF1/CIP1 [53] and the p53/p21 pathway activation [54]. The mechanism underlying HDAC-related apoptosis in tumor cells is still unclear, but it seems to involve the recruitment of Bcl-2 family members. LBH589 (panobinostat) is a nonselective HDAC inhibitor interfering with the deacetylation process. Its activity has been tested in RCC and haematological malignancies. Results from a phase II study [55] conducted in twenty patients who had received at least one prior TKI and one mTOR inhibitor, demonstrated no ORs, although panobinostat was well tolerated. Recently, a phase II trial (NCT01037257) has been opened to investigate its activity in combination with everolimus. 

In the last years, the list of therapeutic targets has widely enlarged, including at present the signal transducer and activator of transcription 3 (STAT 3), Notch homolog 1 translocation-associated (Notch-1), dysmorphic ECs, and nucleolin. Preliminary results from further clinical trials are helpful to assess their role in the future RCC treatment landscape.

STAT 3 is involved in several signaling pathways, regulating cell survival, proliferation, and angiogenesis, and it is aberrantly activated in RCC [56]. In a recent study, the activity of WP1066, a STAT 3 inhibitor, has been tested in vitro in RCC lines and in vivo on murine xenografts; the results obtained show an antiproliferative activity related to a reduction in tubulogenesis [57].

Notch pathway is involved in cell communication and angiogenesis representing a potential target for anticancer therapy. The development of gamma-secretase/Notch signaling pathway inhibitor RO4929097 has led to novel perspectives, and it has been approached in a phase I trial (NCT01141569) in mRCC patients that have failed VEGF/VEGFR therapy. 

The main objective of vascular targeting agents is to inhibit tumor angiogenesis and to avoid the formation of new sprouting vessels. A different approach may be realized by the use of vascular-disrupting agents (VDAs). VDAs recognize and disrupt the already existing tumor vessels by targeting dysmorphic ECs. VDAs include small molecules and ligand-directed agents. Most of the small molecules are tubulin inhibitors, while ligand-directed agents are mainly flavonoids. Their action results in a local production of TNF-alpha and other cytokines. 

Finally, the discovery and development of anticancer aptamers may provide an important contribution to RCC treatment. Aptamers are short DNA, RNA, or peptide oligomers able to assume a specific and stable three-dimensional shape in vivo [58]. The 26-mer DNA aptamer AS1411 is currently undergoing clinical evaluation in acute myeloid leukemia (AML), RCC (NCT00740441), and several other solid tumors. AS1411 is internalized inside tumor cells by specific binding to nucleolin. Nucleolin is involved in several cellular activities, such as mRNA stabilization and ribosome assembly [59]. AS1411 activity seems to be related to its decoy action, which interferes with the stabilization of bcl-2 mRNA by nucleolin, inducing cell death [60].

## 6. Discussion

The advent of targeted therapy has dramatically improved the outcome of mRCC patients. From the approval of sorafenib in 2005, the FDA sequentially approved sunitinib in 2006, temsirolimus in 2007, everolimus, bevacizumab combined with interferon alpha (IFN-*α*), and pazopanib in 2009, and lastly axitinib in 2012. Based on the results obtained by these agents, a large number of drugs have been developed and are currently under evaluation in RCC patients (Table 1). Nevertheless, the efficacy of these agents seems to be influenced by several factors. The analysis of clinical trials described in this review reveals that toxicity and drug resistance are the main driving forces in continuing research and development of novel agents. The toxicity profiles of emerging molecules result mild to moderate, showing relevant grade 3 and grade 4 adverse event rates (range 7–16.1%). Only volociximab showed no serious adverse events. Although there were differences between the frequencies of PR observed in these trials, all investigated agents were characterized by the lack of CR. These negative data should be a strong stimulus for the creation of new therapeutic approaches. 

Primary or acquired resistance to TKIs and mTOR inhibitors has become a major focus for cancer researchers. Primary resistance is less common and seems to be linked to an intrinsic redundancy of tumor available angiogenic signals. On the other hand, acquired resistance is the expression of an “angiogenic switch,” which consists in upregulation of the existing VEGF pathway associated with the recruitment of alternative proangiogenic factors. Current designed preclinical and clinical trials include sequential and combination regimens, aimed at providing a complete blockade of the VEGF pathway. Observing preliminary results, in spite of a mild improvement of patients' outcome, combined therapies have shown a moderate to severe increase of adverse events. Beyond the toxicity reported, the realization of combined regimens is also limited by their high costs that make them feasible in experimental trials but not in clinical practice. 

Moreover, the development of novel effective agents parallels the request of new clinical and molecular predictive and prognostic biomarkers. Their role should be even more relevant if associated with an improved evaluation of tumoral response to antiangiogenic therapy. Finally, an extended knowledge of tumor carcinogenesis process may afford novel tools to optimize treatment regimens. Further clinical trials are still needed to improve the outcome of mRCC patients.

## 7. Conclusion

Data gathered from ongoing trials will surely improve the management of mRCC patients, even if it is difficult to define the relevance of each one's individual contribution. Preliminary results from ongoing trials thus constitute a basis for moderate enthusiasm, but a dramatic improvement of mRCC patient outcomes seems still so far.

## Figures and Tables

**Table 1:** Novel targeted agents currently under evaluation for mRCC.

Agent	Description	Trial ID number	Phase	Design
Brivanib	Dual VEGFR2 and FGFR-1	NCT01253668	II	RCC patients after prior treatment with TKI or bevacizumab
Crizotinib	Alk and c-MET TKI	NCT01524926	II	Patients with solid tumors
BIBF 120	VEGFR 1–3 PDGFR and FGFR TKI	NCT01024920	II	versus sunitinib in untreated mRCC patients
VEGF-Trap	Soluble decoy receptor; derivative of VEGFR1	NCT00357760	II	ccRCC patients after at least 1 prior treatment with TKI
Ridaforolimus	MTORC1 selective inhibitor	NCT01169532	I	In combination with *vorinostat* in patients with solid tumors
		NCT01295632	I	In combination with * MK2206* or *γ*-secretase inhibitor *MK-0752* in patients with advanced solid tumors
MK-2206	AKT inhibitor	NCT01239342	II	Versus everolimus in refractory RCC patients
NVP-BEZ235	Dual PI3K/mTOR inhibitor	NCT01482156	I	In combination with everolimus in patients with advanced solid tumors
GDC-0980	Dual PI3K/mTOR inhibitor	NCT01442090	II	In comparison with *everolimus* in mRCC patients progressed on VEGF-targeted therapy
AMG-386	Ang-1/2 inhibitor	NCT01548482	II	In combination with *temsirolimus* in patients with advanced solid tumors
MDX-1203	Anti-CD70 Ab-drug conjugate	NCT00944905	I	Pretreated ccRCC or B-cell non-Hodgkin's lymphoma
MDX-1411	Anti-CD70 Ab-drug conjugate	NCT00656734	I	ccRCC pts treated with up to 6 prior systemic therapies
SGN-75	Anti-CD70 Ab-drug conjugate	NCT01015911	I	Pretreated ccRCC or B-cell non-Hodgkin's lymphoma
Girentuximab	Chimeric mAb *cG250 *	NCT00087022	III	Adjuvant cG250 versus placebo in pts with ccRCC and high risk of recurrence
cG250-Lu177	Lutetium-177 labeled cG250	NCT00142415	II	pts with advanced and progressive ccRCC
90Y-cG250	Yttrium-90 labeled cG250	NCT00199875	I	pts with advanced and progressive ccRCC
Panitumumab	Anti-EGFR mAb	NCT00425035	II	mRCC pts naïve or after cytokine treatment
Vorinostat	HDAC inhibitor	NCT00278395	II	mRCC pts naïve or after cytokine treatment
RO4929097	*γ*-secretase/Notch inhibitor	NCT01141569	II	ccRCC pts after anti-VEGF and/or mTOR inhibitor and/or immunotherapy failure
AS1411	26-mer DNA aptamer	NCT00740441	II	ccRCC pts after at least 1 prior treatment with TKI
